# Common variants in *GNL3* gene contributed the susceptibility of hand osteoarthritis in Han Chinese population

**DOI:** 10.1038/s41598-022-20287-4

**Published:** 2022-09-27

**Authors:** Xi Wang, Lin Xiao, Zhiyuan Wang, Liqiang Zhi, Qiang Li

**Affiliations:** 1 Department of Knee Joint Surgery, Xi’an Honghui Hospital, Xi’an, Shaanxi China; 2 Department of Hand Surgery, Xi’an Honghui Hospital, No. 555 Youyi East Road, Xi’an, 710054 Shaanxi China

**Keywords:** Risk factors, Predictive markers

## Abstract

Osteoarthritis (OA) is one of the most popular degenerative joint diseases. The nucleolar GTP binding protein 3 (*GNL3*) gene encodes guanine nucleotide binding protein-like 3, which is related in cell proliferation, differentiation, and cell cycle regulation. Our study aimed to examine the contribution of *GNL3* gene polymorphisms to the risk of hand OA and its related clinical features. A total of 3387 study participants including 1160 patients with hand OA and 2227 controls were recruited in this study. Eleven SNPs in *GNL3* gene were selected for genotyping. Genetic association signals were examined using Plink. Relationships between significant SNPs and clinical features of hand OA were also explored. SNP rs11177 was found to be strongly associated with susceptibility of hand OA (*P* = 4.32 × 10^–5^). The minor allele of rs11177 was associated with increased susceptibility of hand OA. In addition, significant associations were also identified between genotypes of rs11177 and clinical features of hand OA patients including K-L grade (*P* < 0.01) and categorized pain scores (*P* < 0.01). Significant eQTL signals for rs11177 on *GNL3* in multiple types of human tissues were also identified in GTEx database. Our results have established the link between *GNL3* gene and susceptibility of hand OA.

## Introduction

Osteoarthritis (OA) is a highly prevalent chronic degenerative joint disease and the most common cause of pain and disability in the world^1^. Approximately 500 million people worldwide suffer from osteoarthritis^2^, and the epidemiological data indicates that more than half of people under the age of 65 are currently affected^3^. The prevalence of OA is affected by many factors, such as gender, age, geographical area of the study performed, genetic factors, occupation, and diet^4^. In general, the estimated prevalence of hand OA are higher than knee and hip OA^4^. Furthermore, hand OA was more popular in women, especially symptomatic osteoarthritis^5^. Genetic and environmental risk factors contribute to this complex disease^6^. Genetic risk factors could contribute to the susceptibility of OA and are also related with clinical outcomes of OA at different stages of the disease course^6^. Compared to knee and hip OA, the heritability of hand OA was reported to be the highest (approximately 60%)^7^. Previous studies have identified some candidate genes for hand OA, such as the *ACAN* gene (encoding an component of the extracellular matrix in cartilaginous tissue) and the *HFE* gene (encoding a protein named homeostatic iron regulator which is associated with hemochromatosis)^8^. However, the underlying pathogenesis of hand OA remains unknown. Therefore, determining the genes responsible for susceptibility to OA, especially hand OA, is desirable.

Genetic association study based on unrelated populations makes it possible to unravel the genetics features of complex disorders. Hence, Multiple susceptibility loci of complex disease have been reported by candidate gene-based association studies^9–11^. Many of the genes including, *GLIS3*, *MS4A13*, *ZC3H11B*, etc^12–14^ have been reported to be related with OA susceptibility and have drawn the attention of researchers. The nucleolar GTP binding protein 3 (*GNL3*) gene encodes a protein which involves in cell proliferation, differentiation and cell cycle regulation^15^. It is associated with chondrocyte differentiation^16^. In a clinical experiment, the gene expression level of *GNL3* was identified to be increased in the synovial tissue and fluid of OA patients^17^. A missense SNP, rs11177, was identified to be strongly associated with hip OA in a recent genome-wide association (GWA) study based on European populations^18^. Furthermore, a previous study in the Han Chinese population reported that variants in the *GNL3* gene contributed to an increased risk for knee OA^19^. A previous study also revealed that GNL3 could upregulate the levels of IL24 and PTN, which would further strengthen the OA development through inducing articular osteocyte apoptosis and angiogenesis^20^. All these results indicated that the nucleolar GTP binding protein 3 might play key roles in the onset and development of knee and hip OA. However, the association between the genetic polymorphisms of *GNL3* gene and hand OA has yet to be investigated in the Han Chinese population. Therefore, the relationship between GNL3 and hand OA is the focus of our study.

In our study, we aimed to investigate the link between *GNL3* gene and hand OA susceptible risk and clinical features in Han Chinese population, which would provide exciting new insights into the etiology of hand OA and could one day be employed as a treatment target.

## Material and methods

### Study subjects

In the study, a total of 3387 patients with hand OA and 2227 controls were collected from Xi’an Honghui Hospital, and all of the participants were unrelated Han Chinese people. hand OA was diagnosed based on clinical examination and radiographic inspection. The distal interphalangeal (DIP), proximal interphalangeal (PIP), thumb interphalangeal (IP) and metacarpophalangeal (MCP) joints were assessed for both hands of the patients by radiographed examinations. According to the Kellgren and Lawrence (K-L) grading standard, affected joints were confirmed for the presence of hand OA. Patients who had more than two finger joints with K-L grade ≥ 2 were classified as hand OA cases. An 11-score pain analog scale (PAS) ranging from 0 to 10 (0, no pain; 10, worst imaginable pain) was utilized to assess the severity of pain for all patients. All controls were free of symptoms of arthritis or other joint-related diseases and other rheumatic diseases. The exclusion criteria for all participants were a history of gout, pseudogout, rheumatoid arthritis, other forms of arthritis, history of hand joint surgery, secondary hand OA, hand joint trauma, and other hand joint diseases caused by chronic inflammatory diseases. Peripheral blood samples were collected from the study subjects and preserved for further genotyping experiments. Clinical characteristics and demographic information of the study subjects were collected from questionnaires and medical records and are shown in Table 1. Each subject signed a written informed consent form. The Ethics Committee of Xi’an Honghui Hospital approved the study with reference B15REA084 dated 2 September 2015, and the study procedures were carried out based on the Declaration of Helsinki (version 2002).

**Table 1:** Characteristics and demographic information of the study subjects.

Variables	Patients with hand OA (N = 1160)	Controls (N = 2227)	Statistics	*P*-Value
**Gender (%)**				
Male	503 (43)	967 (43)		
Female	657 (57)	1260 (57)	χ^2^ = 1.03 × 10^–29^	1.00
Age, years	61.1 ± 7.4	61.4 ± 7.4	*t* = − 1.04	0.30
BMI, kg/m^2^	26.3 ± 1.5	26.2 ± 1.5	*t* = 1.80	0.07
Pain Analog Scale	5.3 ± 2.0	–	–	
**K-L grading (%)**				
KL-2	550 (47)	–	–	–
KL-3	366 (32)	–	–	–
KL-4	244 (21)	–	–	–

Continuous variables were presented by mean ± standard deviation.

### SNP selection and molecular biological experiments

Gene *GNL3* is a relatively short gene and therefore we have extracted all the SNPs with MAF ≥ 0.01 within the whole gene region based on 1000 genome project CHB (Chinese Han Beijing) data. Information of 11 SNPs was extracted for genotyping. The genetic information of these 11 SNPs is summarized in supplemental Table S1. Using DNA extraction kits and following the manufacturer's protocol, genomic DNA was isolated from peripheral blood samples (Axygen Scientific Inc.). The Sequenom MassARRAY platform was used to genotype SNPs. Genotype calls were released using Typer Analyzer. Technicians involved in genotyping experiments were blinded to labels of samples. As a quality control procedure, 5% of the samples were replicated for genotyping and the results were matched for all of the replicated samples.

### Statistical analyses

Demographical and clinical characteristics of the study participants were summarized and compared between patients with hand OA and controls. Hardy–Weinberg equilibrium (HWE) tests were conducted for the 11 selected SNPs in control group as quality control for accuracy of genotyping. Single marker based associations were tested in the modes of genotypes and alleles. Cochran–Armitage tests for trend were applied to testing statistical significance. Linkage disequilibrium (LD) blocks were built for the genotyped SNPs and haplotype-based associations were examined within each LD blocks. In addition to targeting on the susceptibility of hand OA, relationships between significant SNPs and K-L grades and pain scores of patients with hand OA were also explored and the statistical significance were examined by χ^2^ tests. Pain scores were categorized as three levels (level 1: score 1–3, level 2: score 4–6 and level 3: score 7–9). Genetic association analyses were mainly implemented using Plink^21^. LD blocks were visualized using Haploview^22^. To correct for the multiple comparisons, the threshold of *P*-value was 0.0045(0.05/11) in the SNP-based association analyses.

### Bioinformatics analyses

Bioinformatics tools SIFT^23^ and Polyphen2^24^ were used for predicting the functional consequences of non-synonymous changes. GTEx database was utilized for exploring the expression quantitative trait loci (eQTL) patterns of the relevant SNPs on *GNL3* and its surrounding loci based on gene expression data obtained from multiple types of human tissues^25^. The roles of SNPs in regulatory DNA elements were examined using RegulomeDB^26^.

## Results

### Baseline information of the study participants

A total of 3387 subjects comprised of 1160 patients with hand OA and 2227 controls were recruited in this study (Table 1). Distributions for age (*P* = 0.3), gender (*P* = 1.0) and body mass index (*P* = 0.07) between patients with hand OA and controls are in balanced. Average pain scores for patients with hand OA was 5.3. Among patients, 47% of them were classified as KL-2, 32% were classified as KL-3 and 21% were classified as KL-4.

### Genetic association between rs11177 and susceptibility of hand OA

All of the 11 SNPs were in Hardy–Weinberg equilibrium as showed in Supplemental Table S1. The genotypes of a non-synonymous variant, rs11177, were identified to be significantly related with risk of hand OA (Table 2, χ^2^ = 20.10, *P* = 4.32 × 10^–5^, post-hoc power = 75.6%). Similar association patterns were also observed for the alleles of rs11177 with the susceptibility of hand OA (χ^2^ = 19.89, *P* = 8.19 × 10^–6^). Its minor allele, A allele, was related with increased risk of hand OA (OR[95%CI] = 1.26[1.14–1.39]). Supplementary Table S2 contains the complete results of SNP-based association studies. There were a total of two LD blocks constructed (Supplementary Figures S1 and S2). The LD block of rs1108842-rs11177 was found to be related with hand OA susceptibility (Table 3, χ^2^ = 65.60, *P* = 5.69 × 10^–15^). This haplotype based association signal might be originated from rs11177.

**Table 2:** Significant association signal obtained from SNP rs11177.

SNP	Position	Status	Genotypic analyses						Allelic analyses				
			AA	AG	GG	χ^2^	*P*	*P*_add	A	G	χ^2^	OR [95%CI]	*P*
rs11177	3:52687289	Patients	289 (25)	578 (50)	293 (25)				1156 (50)	1164 (50)			
		Controls	429 (19)	1108 (50)	690 (31)	20.10	4.32 × 10^–5^	8.57 × 10^–6^	1966 (44)	2488 (56)	19.89	1.26 [1.14–1.39]	8.19 × 10^–6^

**Table 3:** Results of the haplotype based association analyses.

Locus	Length (kb)	Haplotype	F_A	F_U	χ^2^	DF	*P*	OR [95%CI]^a^	SNPs
H1	1.23	OMNIBUS	–	–	65.60	2	5.69 × 10^–15^		rs1108842|rs11177
		CA	0.49	0.44	14.23	1	0.0002	Ref	rs1108842|rs11177
		CG	0.01	0.04	59.13	1	1.48 × 10^–14^	0.82 [0.74–0.90]	rs1108842|rs11177
		AG	0.50	0.52	1.19	1	0.2763		rs1108842|rs11177
H2	0.74	OMNIBUS	–	–	2.47	2	0.2914		rs13076193|rs6762813
		AT	0.46	0.46	0.01	1	0.9135	Ref	rs13076193|rs6762813
		AC	0.04	0.03	2.44	1	0.1186	1.00 [0.90–1.11]	rs13076193|rs6762813
		CC	0.50	0.51	0.23	1	0.6342		rs13076193|rs6762813

F_A, haplotype frequency in patients with hand OA; F_U, haplotype frequency in controls; DF, degree of freedom.

^a^For H1, the ORs were calculated for CG + AG versus CA. For H2, the ORs were calculated for AC + CC versus AT.

### Relationship between rs11177 and clinical features of hand OA

Significant differences were identified for both K-L grade and categorized PAS of hand OA patients with different genotypes of rs11177 (Table 4). For patients with AA genotypes, 29% of them were classified as KL-2 and 33% as KL-4; while for patients with GG genotypes, 66% of them were classified as KL-2 and 11% as KL-4. The copy number of A alleles were related to more severe symptoms of hand OA in the patients. Similar patterns could also be observed in categorized PAS. For patients with AA genotypes, 9% of them were classified as level 1 in PAS and 55% as level 3; while for patients with GG genotypes, 37% of them were classified as level 1 and 12% as level 3. The copy number of A alleles were associated with higher PAS in patients with hand OA.

**Table 4:** Association between SNP rs11177 and clinical severity and pain analog scale in patients with hand OA.

Clinical scale		AA (N = 289)	AG (N = 578)	GG (N = 293)	χ^2^	*P*
KL grade	KL-2	84 (29)	273 (47)	193 (66)		
	KL-3	111 (38)	187 (32)	68 (23)		
	KL-4	94 (33)	118 (21)	32 (11)	84.96	< 0.01
Pain Analog Scale	Level 1: 1–3	26 (9)	128 (22)	107 (37)		
	Level 2: 4–6	103 (36)	314 (54)	148 (51)		
	Level 3: 7–9	160 (55)	136 (24)	38 (12)	165.02	< 0.01

### Functional effects of SNP r11177

**Figure 1:**
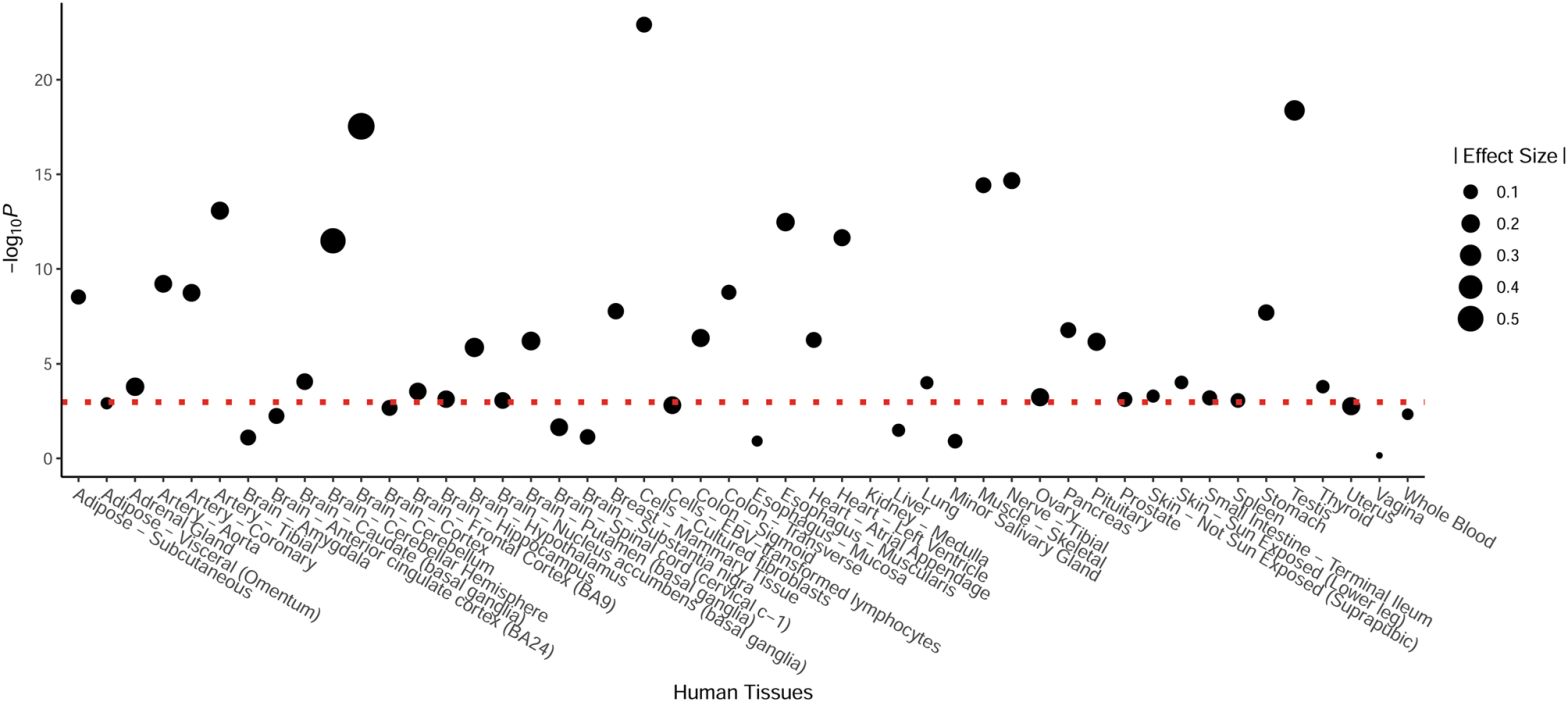
eQTL signals obtained for SNP rs11177 on gene *GNL3* in 47 types of human tissues. Significance threshold for –log*P* was indicated by red dotted line.

SNP rs11177 is classified as “tolerated” in SIFT and “begine” in Polyphen2, which indicates that this SNP might have very limited functional effects on structure of protein encoded by *GNL3* despite of the factor that it is a non-synonymous variant. RS11177 has a RegulomeDB score of 1d. The RegulomeDB score ranges from 1 to 7, and a larger score indicates less important functional consequence. Thus, a score of 1d indicates that this SNP are involved in several genome regulatory process. SNP rs11177 was identified to be a significant eQTL on *GNL3* in multiple types of human tissues (Fig. 1 and Supplementary Table S3). Further investigation showed that rs11177 was not only associated with *GNL3* gene expression but also correlated with the expressions of several other genes located nearby (Supplementary Table S4).

## Discussion

Although a recent study in the Han Chinese individuals has linked the genetic polymorphisms of *GNL3* and knee OA^19^, the link between the *GNL3* gene and hand OA has not yet been examined in the Han Chinese population. In addition, SNP rs11177 has been reported in two independent GWA studies to be associated with OA^18,27^. Nevertheless, both of the previous studies were based on European populations. In the present study, the effect direction of rs11177 was the same as those in the previous studies mentioned above. In this sense, our results could be considered a first evaluation between common variants of the *GNL3* gene and hand OA in the Chinese Han population. Since no evidence has indicated that OA affects different parts of the body and has a different genetic basis, our findings could also be considered a successful replication of the previous GWA significant hit observed in European populations. In addition to susceptibility to hand OA, the association between genetic polymorphisms and clinical features of hand OA were also examined. Both K-L grade and PAS were significantly associated with genotypes of rs11177. A marked dosage dependence pattern was observed. More copy numbers of A alleles of rs11177 were associated with more severe K-L grade and higher PAS score. This finding could shed light on the clinical screening and application of relevant genetic polymorphisms in hand OA in the future.

All of the previous studies (including the present study) focusing on the relationship between rs11177 and OA showed that the A allele is related with an increased susceptibility of OA. In addition, a previous clinical study showed that the gene expression level of *GNL3* was significantly higher in the OA group of synovial tissue and fluid samples^17^. If both findings are true, we could deduce that the A allele should be related to an increased expression level of *GNL3*. However, this finding was not in accordance with the results of our in silico analyses. The A allele was associated with a decreased expression level of *GNL3* in according to the GTEx data. A potential explanation for this inconsistency is that the GTEx data were derived from study participants with unclear clinical status. The expression and eQTL patterns might differ among the tissues of OA patients versus the controls. In addition, although 47 types of human tissues are included in the GTEx database, the target tissue of OA (synovial tissue) is not included. Therefore, the eQTL pattern might be different. Further functional studies are thus needed to unravel the functional mechanisms of the significant genetic markers in *GNL3* to hand OA.

In silico evidence indicates that SNP rs11177 might have functional significance, although further in vitro or in vivo evidence is still needed for validation. Significant eQTL effects of rs11177 on *GNL3* gene expression levels were reported in 34 out of 47 types of human tissues. Although SNP rs11177 is an intronic SNP and thus it might not alter the protein structure encoded by *GNL3*. Evidence in eQTL signals have indicated that this SNP might be related with the gene expression levels of relevant genes. In other words, this SNP might change the gene expression level of relevant genes and in turn influence the hand OA development. In addition, the eQTL effect of SNP rs11177 was not specific to *GNL3*. Further analyses indicated that SNP rs11177 might be related with the expression levels of a couple of other genes located within approximately ± 500 kb from *GNL3*. Among these surrounding genes, *NT5DC2* is of particular interest. NT5DC2 was reported to affect the phosphorylation of tyrosine hydroxylase by regulating its catalytic activity^28^. The genetic polymorphisms of *NT5DC2 gene* has been reported as related with risk of knee OA in a previous GWAS^24^. Although it is out of the scope of the present study, these results are proposing a key question how we should map rs11177 back to the genome. Rs11177 is mapped to *GNL3* according to its physical location, but on the other hand, it could also be mapped to some other surrounding genes such as *NT5DC2* according to its functional relevance. Since both loci have been found to be related with OA susceptibility in previous GWA studies, population based observatory studies might have limited power in addressing this question. Furthermore, given the delicate molecular regulatory mechanisms underlying complex diseases or traits^29–33^, mechanistic validation in animal models would be critical in future studies.

It is worth mentioning some limitations this study suffered. One obvious limitation was that potential population stratification might not be avoided. The present study was implemented based on a candidate gene based study design, therefore it is very difficult to deal with the population stratification through some standard procedures such as principle component analysis. Nevertheless, we have restricted the genetic background of study participants by limiting their immigration history within three generations during the enrollment process. In addition, another potential limitation is that only common variants located within *GNL3* gene region were considered in the present study. However, it has been proved that the genomic regions of ± 10 kb of a gene might contain important regulatory elements.

## Conclusions

To sum up, our results have established the link between a common variant rs11177 in gene *GNL3* and susceptibility of hand OA, and the same polymorphism also associated with the clinical severity in hand OA in a dosage dependence pattern. These results would provide exciting new insights into the etiology of hand OA and could one day be employed as a treatment target.

## Supplementary Information

Supplementary Information.

## Data Availability

The datasets used and/or analyzed during the current study available from the corresponding author on reasonable request.
