# Supplementary material for: Common variants in GNL3 gene contributed the susceptibility of hand osteoarthritis in Han Chinese population
Source: Sci Rep. 2022 Sep 27;12:16110. doi: 10.1038/s41598-022-20287-4 (PMC9515075; doi:10.1038/s41598-022-20287-4)
Supplement: Supplementary file 1 — Supplementary Information. [file 41598_2022_20287_MOESM1_ESM.docx]

***Title:*** Common variants in *GNL3* gene contributed the susceptibility of hand osteoarthritis in Han Chinese population

***Author names and affiliations***: Xi Wang ^1^, Lin Xiao ^1^, Zhiyuan Wang ^1^, Liqiang Zhi ^1^, Qiang Li ^2^.

1 Department of Joint Surgery, Xi'an Honghui Hospital, Xi'an, Shaanxi, China;

2 Department of Hand Surgery, Xi’an Honghui Hospital, Xi'an, Shaanxi, China.

***Corresponding author***:

Qiang Li, Department of Hand Surgery, Xi’an Honghui Hospital, No.555 Youyi East Road, Xi'an, Shaanxi, China, 710054.

Tel: 86-29-88418009; Fax: 86-29-62818386; E-mail: qiangliost@163.com

Supplemental Table S1. Basic information of the 11 genotyped SNPs.

| Chromosome | Position | SNP | Function | A1 | A2 | MAF | MAF(E) | HWE |
| --- | --- | --- | --- | --- | --- | --- | --- | --- |
| 3 | 52686064 | rs1108842 | untranslated-5 | C | A | 0.48 | 0.47 | 0.87 |
| 3 | 52687289 | rs11177 | missense | A | G | 0.46 | 0.40 | 0.70 |
| 3 | 52688432 | rs117150867 | intron | A | G | 0.08 | 0.00 | 1.00 |
| 3 | 52691945 | rs13076193 | intron | A | C | 0.49 | 0.42 | 0.87 |
| 3 | 52689876 | rs183781382 | intron | T | C | 0.07 | 0.00 | 0.62 |
| 3 | 52693241 | rs2289247 | missense | A | G | 0.46 | 0.43 | 0.73 |
| 3 | 52691755 | rs35315313 | intron | T | G | 0.49 | 0.43 | 0.83 |
| 3 | 52688546 | rs35911561 | intron | C | T | 0.07 | 0.10 | 0.87 |
| 3 | 52688319 | rs3774349 | intron | A | C | 0.49 | 0.48 | 0.70 |
| 3 | 52692679 | rs6762813 | intron | T | C | 0.46 | 0.42 | 0.80 |
| 3 | 52691084 | rs75373137 | intron | C | T | 0.07 | 0.00 | 0.45 |

MAF: minor allele frequency; MAF(E): minor allele frequency in Europeans in 1000 genome data. HWE: *P* values for Hardy-Weinberg equilibrium test. A1: minor allele; A2: major allele.

Supplemental Table S2. Full results of the single marker based association analyses.

| CHR | SNP | A1 | A2 | TEST | AFF | UNAFF | χ^2^ | DF | *P* |
| --- | --- | --- | --- | --- | --- | --- | --- | --- | --- |
| 3 | rs1108842 | C | A | GENO | 272/587/301 | 519/1108/600 | 0.40 | 2 | 0.82 |
| 3 | rs1108842 | C | A | TREND | 1131/1189 | 2146/2308 | 0.20 | 1 | 0.66 |
| 3 | rs11177 | A | G | GENO | 289/578/293 | 429/1108/690 | 20.10 | 2 | 4.32×10^-5^ |
| 3 | rs11177 | A | G | TREND | 1156/1164 | 1966/2488 | 19.89 | 1 | 8.19×10^-6^ |
| 3 | rs3774349 | A | C | GENO | 273/590/297 | 521/1122/584 | 0.15 | 2 | 0.93 |
| 3 | rs3774349 | A | C | TREND | 1136/1184 | 2164/2290 | 0.09 | 1 | 0.77 |
| 3 | rs117150867 | A | G | GENO | 7/162/991 | 14/325/1888 | 0.26 | 2 | 0.88 |
| 3 | rs117150867 | A | G | TREND | 176/2144 | 353/4101 | 0.24 | 1 | 0.62 |
| 3 | rs35911561 | C | T | GENO | 10/148/1002 | 10/277/1940 | 2.31 | 2 | 0.31 |
| 3 | rs35911561 | C | T | TREND | 168/2152 | 297/4157 | 0.77 | 1 | 0.38 |
| 3 | rs183781382 | T | C | GENO | 5/154/1001 | 12/281/1934 | 0.46 | 2 | 0.79 |
| 3 | rs183781382 | T | C | TREND | 164/2156 | 305/4149 | 0.12 | 1 | 0.73 |
| 3 | rs75373137 | C | T | GENO | 7/155/998 | 15/306/1906 | 0.16 | 2 | 0.93 |
| 3 | rs75373137 | C | T | TREND | 169/2151 | 336/4118 | 0.15 | 1 | 0.70 |
| 3 | rs35315313 | T | G | GENO | 274/591/295 | 526/1119/582 | 0.22 | 2 | 0.90 |
| 3 | rs35315313 | T | G | TREND | 1139/1181 | 2171/2283 | 0.08 | 1 | 0.78 |
| 3 | rs13076193 | A | C | GENO | 280/589/291 | 536/1118/573 | 0.18 | 2 | 0.92 |
| 3 | rs13076193 | A | C | TREND | 1149/1171 | 2190/2264 | 0.08 | 1 | 0.78 |
| 3 | rs6762813 | T | C | GENO | 248/576/336 | 476/1100/651 | 0.03 | 2 | 0.99 |
| 3 | rs6762813 | T | C | TREND | 1072/1248 | 2052/2402 | 0.01 | 1 | 0.92 |
| 3 | rs2289247 | A | G | GENO | 248/582/330 | 485/1100/642 | 0.19 | 2 | 0.91 |
| 3 | rs2289247 | A | G | TREND | 1078/1242 | 2070/2384 | 5.60×10^-5^ | 1 | 0.99 |

Supplemental Table S3. eQTL signals obtained for rs11177 on *GNL3* in 47 types of human tissues.

| Gene | SNP | *P*-Value | NES | T-statistic | Tissue |
| --- | --- | --- | --- | --- | --- |
| *GNL3* | rs11177 | 1.20×10^-23^ | -0.13 | -11.00 | Cells - Cultured fibroblasts |
| *GNL3* | rs11177 | 4.10×10^-19^ | -0.27 | -9.70 | Testis |
| *GNL3* | rs11177 | 2.80×10^-18^ | -0.55 | -9.80 | Brain - Cerebellum |
| *GNL3* | rs11177 | 2.10×10^-15^ | -0.16 | -8.20 | Nerve - Tibial |
| *GNL3* | rs11177 | 3.70×10^-15^ | -0.13 | -8.10 | Muscle - Skeletal |
| *GNL3* | rs11177 | 8.20×10^-14^ | -0.19 | -7.70 | Artery - Tibial |
| *GNL3* | rs11177 | 3.30×10^-13^ | -0.20 | -7.50 | Esophagus - Muscularis |
| *GNL3* | rs11177 | 2.20×10^-12^ | -0.16 | -7.30 | Heart - Left Ventricle |
| *GNL3* | rs11177 | 3.20×10^-12^ | -0.48 | -7.70 | Brain - Cerebellar Hemisphere |
| *GNL3* | rs11177 | 6.00×10^-10^ | -0.18 | -6.40 | Artery - Aorta |
| *GNL3* | rs11177 | 1.70×10^-9^ | -0.11 | -6.20 | Colon - Transverse |
| *GNL3* | rs11177 | 1.80×10^-9^ | -0.18 | -6.40 | Artery - Coronary |
| *GNL3* | rs11177 | 3.00×10^-9^ | -0.11 | -6.00 | Adipose - Subcutaneous |
| *GNL3* | rs11177 | 1.70×10^-8^ | -0.14 | -5.80 | Breast - Mammary Tissue |
| *GNL3* | rs11177 | 2.00×10^-8^ | -0.14 | -5.80 | Stomach |
| *GNL3* | rs11177 | 1.70×10^-7^ | -0.13 | -5.40 | Pancreas |
| *GNL3* | rs11177 | 4.40×10^-7^ | -0.19 | -5.20 | Colon - Sigmoid |
| *GNL3* | rs11177 | 5.60×10^-7^ | -0.13 | -5.10 | Heart - Atrial Appendage |
| *GNL3* | rs11177 | 6.40×10^-7^ | -0.21 | -5.20 | Brain - Putamen (basal ganglia) |
| *GNL3* | rs11177 | 7.00×10^-7^ | -0.19 | -5.10 | Pituitary |
| *GNL3* | rs11177 | 1.40×10^-6^ | -0.23 | -5.10 | Brain - Hypothalamus |
| *GNL3* | rs11177 | 8.60×10^-5^ | -0.15 | -4.00 | Brain - Caudate (basal ganglia) |
| *GNL3* | rs11177 | 9.50×10^-5^ | -0.08 | -3.90 | Skin - Sun Exposed (Lower leg) |
| *GNL3* | rs11177 | 9.90×10^-5^ | -0.07 | -3.90 | Lung |
| *GNL3* | rs11177 | 0.0002 | -0.20 | -3.90 | Adrenal Gland |
| *GNL3* | rs11177 | 0.0002 | -0.08 | -3.80 | Thyroid |
| *GNL3* | rs11177 | 0.0003 | -0.17 | -3.70 | Brain - Frontal Cortex (BA9) |
| *GNL3* | rs11177 | 0.0005 | -0.07 | -3.50 | Skin - Not Sun Exposed (Suprapubic) |
| *GNL3* | rs11177 | 0.0006 | -0.19 | -3.50 | Ovary |
| *GNL3* | rs11177 | 0.0006 | -0.11 | -3.50 | Small Intestine - Terminal Ileum |
| *GNL3* | rs11177 | 0.0007 | -0.17 | -3.50 | Brain - Hippocampus |
| *GNL3* | rs11177 | 0.0008 | -0.11 | -3.40 | Prostate |
| *GNL3* | rs11177 | 0.0009 | -0.15 | -3.40 | Brain - Nucleus accumbens (basal ganglia) |
| *GNL3* | rs11177 | 0.0009 | -0.10 | -3.40 | Spleen |
| *GNL3* | rs11177 | 0.0012 | -0.06 | -3.30 | Adipose - Visceral (Omentum) |
| *GNL3* | rs11177 | 0.0015 | -0.18 | -3.20 | Cells - EBV-transformed lymphocytes |
| *GNL3* | rs11177 | 0.0017 | -0.19 | -3.20 | Uterus |
| *GNL3* | rs11177 | 0.0021 | -0.13 | -3.10 | Brain - Cortex |
| *GNL3* | rs11177 | 0.0046 | -0.05 | -2.80 | Whole Blood |
| *GNL3* | rs11177 | 0.0056 | -0.13 | -2.80 | Brain - Anterior cingulate cortex (BA24) |
| *GNL3* | rs11177 | 0.0220 | -0.18 | -2.30 | Brain - Spinal cord (cervical c-1) |
| *GNL3* | rs11177 | 0.0320 | -0.07 | -2.20 | Liver |
| *GNL3* | rs11177 | 0.0720 | -0.12 | -1.80 | Brain - Substantia nigra |
| *GNL3* | rs11177 | 0.0770 | -0.13 | -1.80 | Brain - Amygdala |
| *GNL3* | rs11177 | 0.1200 | -0.04 | -1.60 | Esophagus - Mucosa |
| *GNL3* | rs11177 | 0.1200 | -0.10 | -1.60 | Minor Salivary Gland |
| *GNL3* | rs11177 | 0.6900 | 0.02 | 0.40 | Vagina |

NES: normalized effect size.

Supplemental Table S4. eQTL signals that achieved genome-wide significance for SNP rs11177 on multiple genes.

| Gene Symbol | SNP | *P*-Value | NES | Tissue |
| --- | --- | --- | --- | --- |
| *DNAH1* | rs11177 | 2.30E-04 | 0.07 | Cells - Cultured fibroblasts |
| *GLT8D1* | rs11177 | 1.30E-06 | 0.11 | Thyroid |
| *GLYCTK* | rs11177 | 4.10E-11 | -0.24 | Nerve - Tibial |
| *GLYCTK* | rs11177 | 2.00E-10 | -0.16 | Whole Blood |
| *GLYCTK* | rs11177 | 1.30E-09 | -0.20 | Thyroid |
| *GLYCTK* | rs11177 | 1.90E-09 | -0.20 | Esophagus - Mucosa |
| *GLYCTK* | rs11177 | 1.30E-08 | -0.17 | Lung |
| *GLYCTK* | rs11177 | 2.40E-06 | -0.33 | Brain - Cerebellum |
| *GLYCTK* | rs11177 | 3.50E-06 | -0.31 | Pituitary |
| *GLYCTK* | rs11177 | 8.10E-06 | -0.15 | Esophagus - Muscularis |
| *GLYCTK* | rs11177 | 9.70E-06 | -0.29 | Spleen |
| *GLYCTK* | rs11177 | 7.90E-05 | -0.10 | Colon - Transverse |
| *GLYCTK* | rs11177 | 1.70E-04 | -0.17 | Esophagus - Gastroesophageal Junction |
| *GLYCTK-AS1* | rs11177 | 2.70E-10 | 0.32 | Testis |
| *GNL3* | rs11177 | 1.20E-23 | -0.13 | Cells - Cultured fibroblasts |
| *GNL3* | rs11177 | 4.10E-19 | -0.27 | Testis |
| *GNL3* | rs11177 | 2.80E-18 | -0.55 | Brain - Cerebellum |
| *GNL3* | rs11177 | 2.10E-15 | -0.16 | Nerve - Tibial |
| *GNL3* | rs11177 | 3.70E-15 | -0.13 | Muscle - Skeletal |
| *GNL3* | rs11177 | 8.20E-14 | -0.19 | Artery - Tibial |
| *GNL3* | rs11177 | 3.30E-13 | -0.20 | Esophagus - Muscularis |
| *GNL3* | rs11177 | 2.20E-12 | -0.16 | Heart - Left Ventricle |
| *GNL3* | rs11177 | 3.20E-12 | -0.48 | Brain - Cerebellar Hemisphere |
| *GNL3* | rs11177 | 6.00E-10 | -0.18 | Artery - Aorta |
| *GNL3* | rs11177 | 1.70E-09 | -0.11 | Colon - Transverse |
| *GNL3* | rs11177 | 1.80E-09 | -0.18 | Artery - Coronary |
| *GNL3* | rs11177 | 3.00E-09 | -0.11 | Adipose - Subcutaneous |
| *GNL3* | rs11177 | 6.20E-09 | -0.20 | Esophagus - Gastroesophageal Junction |
| *GNL3* | rs11177 | 1.70E-08 | -0.14 | Breast - Mammary Tissue |
| *GNL3* | rs11177 | 2.00E-08 | -0.14 | Stomach |
| *GNL3* | rs11177 | 1.70E-07 | -0.13 | Pancreas |
| *GNL3* | rs11177 | 4.40E-07 | -0.19 | Colon - Sigmoid |
| *GNL3* | rs11177 | 5.60E-07 | -0.13 | Heart - Atrial Appendage |
| *GNL3* | rs11177 | 6.40E-07 | -0.21 | Brain - Putamen (basal ganglia) |
| *GNL3* | rs11177 | 7.00E-07 | -0.19 | Pituitary |
| *GNL3* | rs11177 | 1.40E-06 | -0.23 | Brain - Hypothalamus |
| *GNL3* | rs11177 | 8.60E-05 | -0.15 | Brain - Caudate (basal ganglia) |
| *GNL3* | rs11177 | 9.50E-05 | -0.08 | Skin - Sun Exposed (Lower leg) |
| *GNL3* | rs11177 | 9.90E-05 | -0.07 | Lung |
| *GNL3* | rs11177 | 1.60E-04 | -0.08 | Thyroid |
| *ITIH1* | rs11177 | 2.60E-09 | -0.29 | Nerve - Tibial |
| *ITIH4* | rs11177 | 1.70E-31 | 0.63 | Artery - Aorta |
| *ITIH4* | rs11177 | 4.10E-29 | 0.51 | Artery - Tibial |
| *ITIH4* | rs11177 | 3.80E-25 | 0.30 | Whole Blood |
| *ITIH4* | rs11177 | 6.60E-25 | 0.37 | Thyroid |
| *ITIH4* | rs11177 | 9.10E-24 | 0.42 | Cells - Cultured fibroblasts |
| *ITIH4* | rs11177 | 1.80E-19 | 0.38 | Lung |
| *ITIH4* | rs11177 | 4.90E-17 | 0.30 | Skin - Not Sun Exposed (Suprapubic) |
| *ITIH4* | rs11177 | 5.50E-14 | 0.26 | Nerve - Tibial |
| *ITIH4* | rs11177 | 2.60E-13 | 0.29 | Esophagus - Mucosa |
| *ITIH4* | rs11177 | 3.00E-13 | 0.28 | Breast - Mammary Tissue |
| *ITIH4* | rs11177 | 7.90E-13 | 0.17 | Muscle - Skeletal |
| *ITIH4* | rs11177 | 2.50E-12 | 0.20 | Adipose - Subcutaneous |
| *ITIH4* | rs11177 | 3.00E-11 | 0.39 | Artery - Coronary |
| *ITIH4* | rs11177 | 5.00E-11 | 0.20 | Skin - Sun Exposed (Lower leg) |
| *ITIH4* | rs11177 | 1.10E-10 | 0.21 | Colon - Transverse |
| *ITIH4* | rs11177 | 7.90E-10 | 0.33 | Heart - Atrial Appendage |
| *ITIH4* | rs11177 | 3.90E-09 | 0.46 | Brain - Hypothalamus |
| *ITIH4* | rs11177 | 5.50E-09 | 0.44 | Brain - Cortex |
| *ITIH4* | rs11177 | 2.60E-08 | 0.21 | Adipose - Visceral (Omentum) |
| *ITIH4* | rs11177 | 6.40E-08 | 0.17 | Esophagus - Muscularis |
| *ITIH4* | rs11177 | 8.00E-08 | 0.39 | Pituitary |
| *ITIH4* | rs11177 | 3.30E-07 | 0.51 | Cells - EBV-transformed lymphocytes |
| *ITIH4* | rs11177 | 5.40E-07 | 0.47 | Brain - Amygdala |
| *ITIH4* | rs11177 | 1.30E-06 | 0.34 | Brain - Caudate (basal ganglia) |
| *ITIH4* | rs11177 | 1.40E-06 | 0.30 | Brain - Putamen (basal ganglia) |
| *ITIH4* | rs11177 | 2.10E-06 | 0.24 | Small Intestine - Terminal Ileum |
| *ITIH4* | rs11177 | 4.40E-06 | -0.27 | Pancreas |
| *ITIH4* | rs11177 | 6.80E-06 | 0.28 | Spleen |
| *ITIH4* | rs11177 | 1.00E-05 | 0.22 | Heart - Left Ventricle |
| *ITIH4* | rs11177 | 3.30E-05 | 0.25 | Testis |
| *ITIH4* | rs11177 | 3.70E-05 | 0.29 | Brain - Nucleus accumbens (basal ganglia) |
| *ITIH4* | rs11177 | 4.90E-05 | 0.17 | Esophagus - Gastroesophageal Junction |
| *MUSTN1* | rs11177 | 4.60E-18 | 0.42 | Artery - Aorta |
| *MUSTN1* | rs11177 | 3.20E-11 | 0.18 | Whole Blood |
| *NEK4* | rs11177 | 2.30E-21 | -0.27 | Thyroid |
| *NEK4* | rs11177 | 2.00E-18 | -0.25 | Skin - Sun Exposed (Lower leg) |
| *NEK4* | rs11177 | 6.00E-15 | -0.24 | Nerve - Tibial |
| *NEK4* | rs11177 | 2.90E-12 | -0.20 | Artery - Tibial |
| *NEK4* | rs11177 | 2.40E-11 | -0.17 | Cells - Cultured fibroblasts |
| *NEK4* | rs11177 | 1.40E-10 | -0.22 | Adipose - Visceral (Omentum) |
| *NEK4* | rs11177 | 5.40E-10 | -0.20 | Esophagus - Mucosa |
| *NEK4* | rs11177 | 5.40E-10 | -0.25 | Breast - Mammary Tissue |
| *NEK4* | rs11177 | 7.40E-10 | -0.22 | Colon - Sigmoid |
| *NEK4* | rs11177 | 7.50E-10 | -0.20 | Esophagus - Gastroesophageal Junction |
| *NEK4* | rs11177 | 1.60E-09 | -0.13 | Muscle - Skeletal |
| *NEK4* | rs11177 | 3.00E-09 | -0.20 | Adipose - Subcutaneous |
| *NEK4* | rs11177 | 4.40E-09 | -0.14 | Esophagus - Muscularis |
| *NEK4* | rs11177 | 5.00E-09 | -0.21 | Heart - Atrial Appendage |
| *NEK4* | rs11177 | 7.80E-08 | -0.13 | Lung |
| *NEK4* | rs11177 | 1.00E-07 | -0.42 | Minor Salivary Gland |
| *NEK4* | rs11177 | 2.50E-07 | -0.21 | Brain - Cerebellar Hemisphere |
| *NEK4* | rs11177 | 4.30E-07 | -0.17 | Colon - Transverse |
| *NEK4* | rs11177 | 1.20E-06 | -0.19 | Spleen |
| *NEK4* | rs11177 | 2.60E-06 | -0.12 | Heart - Left Ventricle |
| *NEK4* | rs11177 | 3.30E-06 | -0.22 | Pancreas |
| *NEK4* | rs11177 | 2.70E-05 | -0.19 | Brain - Cerebellum |
| *NEK4* | rs11177 | 4.10E-05 | -0.19 | Pituitary |
| *NEK4* | rs11177 | 4.30E-05 | -0.12 | Skin - Not Sun Exposed (Suprapubic) |
| *NT5DC2* | rs11177 | 3.70E-35 | -0.29 | Skin - Sun Exposed (Lower leg) |
| *NT5DC2* | rs11177 | 5.10E-24 | -0.23 | Skin - Not Sun Exposed (Suprapubic) |
| *NT5DC2* | rs11177 | 4.80E-23 | -0.27 | Thyroid |
| *NT5DC2* | rs11177 | 6.20E-19 | -0.31 | Colon - Transverse |
| *NT5DC2* | rs11177 | 9.20E-17 | -0.27 | Testis |
| *NT5DC2* | rs11177 | 3.80E-16 | -0.20 | Whole Blood |
| *NT5DC2* | rs11177 | 4.30E-13 | -0.21 | Nerve - Tibial |
| *NT5DC2* | rs11177 | 6.00E-13 | -0.29 | Spleen |
| *NT5DC2* | rs11177 | 1.40E-10 | 0.25 | Heart - Atrial Appendage |
| *NT5DC2* | rs11177 | 4.60E-10 | -0.11 | Cells - Cultured fibroblasts |
| *NT5DC2* | rs11177 | 8.30E-10 | -0.21 | Artery - Aorta |
| *NT5DC2* | rs11177 | 2.00E-09 | -0.31 | Colon - Sigmoid |
| *NT5DC2* | rs11177 | 8.00E-08 | -0.28 | Small Intestine - Terminal Ileum |
| *NT5DC2* | rs11177 | 9.10E-08 | -0.15 | Lung |
| *NT5DC2* | rs11177 | 6.20E-07 | -0.16 | Artery - Tibial |
| *NT5DC2* | rs11177 | 6.40E-07 | -0.15 | Adipose - Visceral (Omentum) |
| *NT5DC2* | rs11177 | 7.70E-07 | -0.39 | Cells - EBV-transformed lymphocytes |
| *NT5DC2* | rs11177 | 1.50E-05 | -0.20 | Pancreas |
| *NT5DC2* | rs11177 | 4.00E-05 | -0.14 | Breast - Mammary Tissue |
| *NT5DC2* | rs11177 | 5.70E-05 | -0.14 | Adipose - Subcutaneous |
| *NT5DC2* | rs11177 | 7.40E-05 | 0.17 | Heart - Left Ventricle |
| *PBRM1* | rs11177 | 1.40E-55 | 0.43 | Thyroid |
| *PBRM1* | rs11177 | 2.10E-25 | 0.17 | Artery - Tibial |
| *PBRM1* | rs11177 | 3.80E-06 | -0.09 | Cells - Cultured fibroblasts |
| *POC1A* | rs11177 | 6.10E-06 | -0.31 | Brain - Putamen (basal ganglia) |
| *PPM1M* | rs11177 | 1.30E-07 | -0.31 | Brain - Cerebellar Hemisphere |
| *PPM1M* | rs11177 | 1.90E-07 | -0.37 | Brain - Cerebellum |
| *PPM1M* | rs11177 | 4.70E-05 | -0.11 | Muscle - Skeletal |
| *PPM1M* | rs11177 | 1.10E-04 | -0.10 | Heart - Left Ventricle |
| *PPM1M* | rs11177 | 1.80E-04 | -0.09 | Nerve - Tibial |
| *PPM1M* | rs11177 | 2.50E-04 | -0.10 | Artery - Tibial |
| *RFT1* | rs11177 | 1.20E-04 | -0.10 | Cells - Cultured fibroblasts |
| *RP11-168J18.6* | rs11177 | 2.90E-04 | 0.17 | Cells - Cultured fibroblasts |
| *RP11-168J18.6* | rs11177 | 4.40E-04 | 0.15 | Thyroid |
| *RP11-894J14.2* | rs11177 | 1.50E-06 | 0.23 | Artery - Tibial |
| *RP5-1157M23.2* | rs11177 | 1.90E-04 | -0.22 | Skin - Not Sun Exposed (Suprapubic) |
| *RP5-1157M23.2* | rs11177 | 4.40E-04 | -0.13 | Cells - Cultured fibroblasts |
| *RP5-966M1.5* | rs11177 | 3.50E-05 | -0.43 | Brain - Cerebellar Hemisphere |
| *RP5-966M1.7* | rs11177 | 1.50E-14 | -0.60 | Brain - Cerebellum |
| *RP5-966M1.7* | rs11177 | 8.50E-08 | -0.42 | Brain - Cerebellar Hemisphere |
| *RP5-966M1.7* | rs11177 | 4.40E-05 | -0.32 | Brain - Cortex |
| *RP5-966M1.7* | rs11177 | 7.00E-05 | -0.45 | Brain - Putamen (basal ganglia) |
| *RP5-966M1.7* | rs11177 | 4.50E-04 | -0.23 | Cells - Cultured fibroblasts |
| *SERBP1P3* | rs11177 | 4.20E-07 | 0.26 | Colon - Sigmoid |
| *SFMBT1* | rs11177 | 6.70E-06 | 0.11 | Whole Blood |
| *SFMBT1* | rs11177 | 3.40E-05 | 0.16 | Heart - Atrial Appendage |
| *SPCS1* | rs11177 | 3.30E-05 | 0.20 | Brain - Frontal Cortex (BA9) |
| *SPCS1* | rs11177 | 8.90E-05 | 0.08 | Muscle - Skeletal |
| *TMEM110* | rs11177 | 3.00E-15 | 0.32 | Artery - Aorta |
| *TMEM110* | rs11177 | 1.50E-11 | 0.14 | Whole Blood |
| *TMEM110* | rs11177 | 7.70E-11 | 0.19 | Muscle - Skeletal |
| *TMEM110* | rs11177 | 3.60E-08 | 0.20 | Lung |
| *TMEM110* | rs11177 | 1.30E-06 | 0.17 | Thyroid |
| *TMEM110* | rs11177 | 2.80E-05 | 0.14 | Nerve - Tibial |
| *TMEM110* | rs11177 | 3.40E-05 | 0.12 | Adipose - Subcutaneous |
| *WDR82* | rs11177 | 4.00E-07 | 0.10 | Cells - Cultured fibroblasts |

NES: normalized effect size.

**Supplemental Figures**


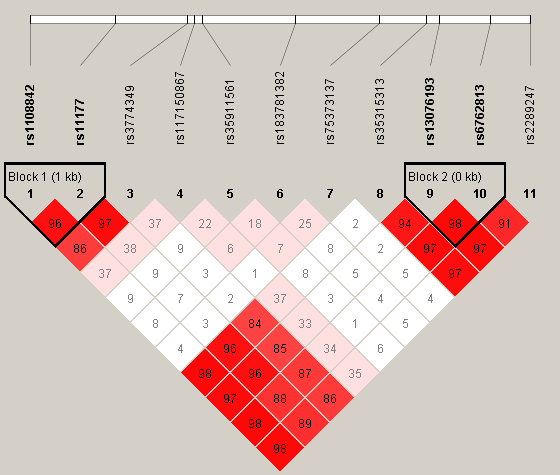


Supplemental Figure S1. Linkage disequilibrium structure for the 11 genotyped SNPs. Values of D’ were indicated in each cell.


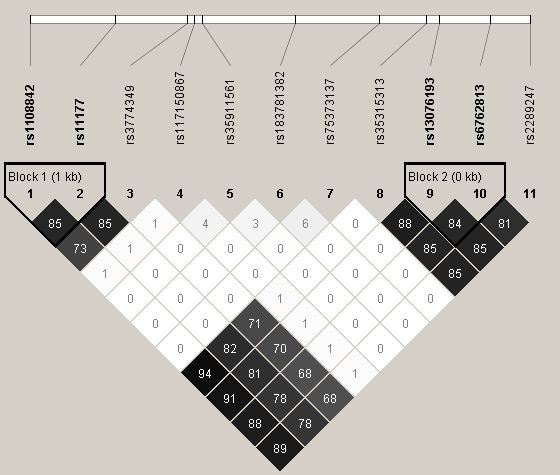


Supplementary Figure S2. Linkage disequilibrium structure for the 11 genotyped SNPs. Values of R^2^ were indicated in each cell.
